# Growth kinetics of amyloid-like fibrils: An integrated atomistic simulation and continuum theory approach

**DOI:** 10.1093/pnasnexus/pgae045

**Published:** 2024-02-01

**Authors:** Ruoyao Zhang, Sharareh Jalali, Cristiano Luis Dias, Mikko P Haataja

**Affiliations:** Department of Mechanical and Aerospace Engineering, Princeton University, Princeton, NJ 08544, USA; Department of Physics, New Jersey Institute of Technology, Newark, NJ 07102, USA; Department of Physics, New Jersey Institute of Technology, Newark, NJ 07102, USA; Department of Mechanical and Aerospace Engineering, Princeton University, Princeton, NJ 08544, USA; Princeton Materials Institute, Princeton University, Princeton, NJ 08544, USA

**Keywords:** amyloid fibrils, fibril growth, molecular dynamics, continuum theory

## Abstract

Amyloid fibrils have long been associated with many neurodegenerative diseases. The conventional picture of the formation and proliferation of fibrils from unfolded proteins comprises primary and secondary nucleation of oligomers followed by elongation and fragmentation thereof. In this work, we first employ extensive all-atom molecular dynamics (MD) simulations of short peptides to investigate the governing processes of fibril growth at the molecular scale. We observe that the peptides in the bulk solution can bind onto and subsequently diffuse along the fibril surface, which leads to fibril elongation via either bulk- or surface-mediated docking mechanisms. Then, to guide the quantitative interpretation of these observations and to provide a more comprehensive picture of the growth kinetics of single fibrils, a continuum model which incorporates the key processes observed in the MD simulations is formulated. The model is employed to investigate how relevant physical parameters affect the kinetics of fibril growth and identify distinct growth regimes. In particular, it is shown that fibrils which strongly bind peptides may undergo a transient exponential growth phase in which the entire fibril surface effectively acts as a sink for peptides. We also demonstrate how the relevant model parameters can be estimated from the MD trajectories. Our results provide compelling evidence that the overall fibril growth rates are determined by both bulk and surface peptide fluxes, thereby contributing to a more fundamental understanding of the growth kinetics of amyloid-like fibrils.

**Significance Statement**

Abnormal deposits of proteins in tissues/organs in the form of amyloid fibrils have been associated with more than 20 degenerative diseases. In this work, we combine extensive, unbiased all-atom simulations with continuum theory to elucidate the governing processes of amyloid-like fibril growth of a short peptide. Our simulations reveal a new pathway for growth wherein peptides bind to the fibril surface, execute effectively 1D diffusion and subsequently become subsumed into the fibril tips. Using key parameters extracted from the all-atom simulations, we demonstrate that this pathway can contribute significantly to the overall fibril elongation kinetics. More broadly, our findings help to shed light on the fundamental processes controlling the amyloid load in diseases.

## Introduction

Amyloid fibrils are supramolecular structures formed during the self-assembly of *β*-sheet-rich proteins such as amyloid-*β* (A*β*), tau, amylin, and *α*-synuclein (1, 2). Their presence in human tissues is often indicative of diseases such as Alzheimer’s, Parkinson’s, and type 2 diabetes (3, 4). Three key mechanisms have been associated with the formation of these structures, namely the nucleation of fibril seeds via primary and secondary processes, and the growth of these seeds into micrometer long fibrils (5, 6). Proteins incorporated into fibrils typically bind to the fibril ends sequentially, one peptide at the time (7). These observations have sparked great interest in developing protocols aimed at controlling the fibril load in diseases (8). However, the molecular mechanisms and pathways enabling fibril growth are poorly understood at the moment; in particular, effects of the fibril surface—which plays a critical role in secondary nucleation—have remained murky at the molecular level (9). Adding to the complexity of the problem, fibril nucleation and growth are not limited to bulk solutions; they may also take place along or within membranes, causing distortion and leakage (10–12). Importantly, a more fundamental understanding of the key mechanisms associated with fibril growth requires the development and application of complementary methods capable of bridging multiple length and time scales (13, 14).

The mechanism by which a peptide binds to the fibril ends accounting for its growth is often described by a two-step dock-and-lock process (8). The *dock* phase describes the process by which peptides land on the fibril tip, while the *lock* phase accounts for the structural rearrangements required for the peptides to adopt the conformation imposed by the fibril template. Two pathways have been envisaged for the locking process wherein docked proteins undergo random conformational changes (constrained only by steric interactions with the tip) or are driven by side chain interactions with the fibril tip (15, 16).

With regard to docking, it is usually assumed that peptides land onto the fibril tip from the solution, corresponding to *bulk-docking* (17–20). In recent years, however, it has become increasingly clear that the fibril surface plays an important role in the formation of amyloid fibrils. In particular, most oligomers which are poised to either form new fibrils or contribute to existing ones are catalyzed at fibril surfaces via secondary nucleation processes (6). This may emerge from nonspecific binding of proteins in solution with the fibril surface (16, 21, 22). Furthermore, in implicit solvent simulations, A*β* proteins were observed to adsorb onto and diffuse along the fibril surface (23). Recently, nucleation and growth of amyloid fibrils were studied via explicit solvent simulations, highlighting the role of the fibril surface (24). It is thus conceivable that such processes may facilitate an alternative docking pathway—*surface-docking*—where adsorbed proteins navigate around the fibril edge and lock onto the fibril tip. Since diffusion along the fibril surface is effectively 1D, this docking pathway may contribute significantly to fibril elongation. Indeed, as will be shown below, our work directly challenges the conventional view of fibril growth via bulk-docking by highlighting and quantifying the role of the fibril surface.

To this end, docking pathways are first investigated using unbiased all-atom molecular dynamics (MD) simulations of large systems comprising an amyloid fibril, a peptide and water molecules. We consider a short amphipathic peptide that mimics the alternating stretches of nonpolar and polar amino acids in the sequence of amyloid proteins. Both bulk-docking and surface-docking processes at the fibril tip are observed in the MD simulations. To guide the quantitative interpretation of these observations and provide a more comprehensive picture of the growth kinetics of single fibrils, a continuum model which incorporates the key processes observed in our all-atom simulations is formulated. The model is then employed to investigate how relevant physical parameters affect the kinetics of fibril growth and identify distinct growth regimes. In particular, it is shown that fibrils which strongly bind peptides may undergo a transient exponential growth phase in which the entire fibril surface effectively acts as a sink for peptides. We also demonstrate how the relevant model parameters can be estimated from the MD trajectories. Finally, we anticipate that our approach may eventually facilitate the identification of specific amyloid fibril growth pathways that can be targeted in diseases.

## Bulk- and surface-mediated fibril growth: MD simulations

**Fig. 1.**
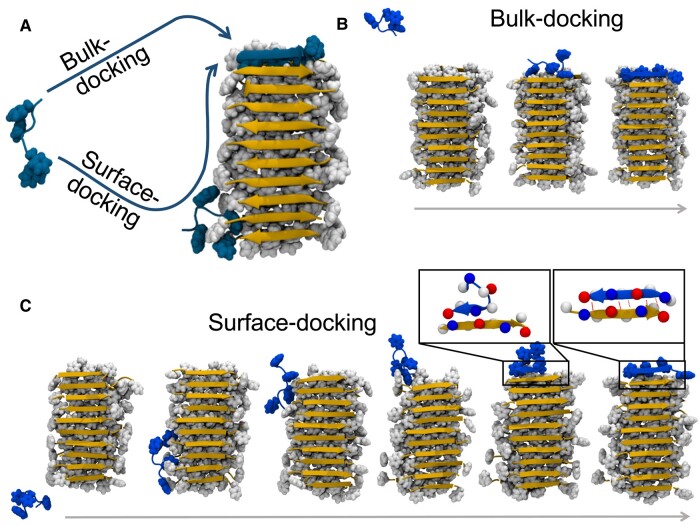
A) Schematic illustration of the observed pathways involved in fibril growth. Representative trajectories of B) bulk- and C) surface-docking pathways where the peptide (in blue) initially in solution binds to fibril tip and surface, respectively. In the surface-docking pathway (panel C), the peptide adsorbs onto the surface and is incorporated into the fibril tip without loss of contact with the surface. Locking is highlighted in zoomed regions of panel C where lysine and glutamic acid residues are represented with blue and red beads, respectively. Phenylalanine and backbone of peptides in the fibril are represented in white and orange, respectively.

To investigate the different pathways accounting for docking, all-atom simulations were performed with a peptide initially placed randomly in the simulation box with a 2 nm minimum separation between the peptide and the preformed fibril. The latter consists of two laminated antiparallel *β*-sheets made of 10 peptides each (cf. Fig. S1B). Due to the stochastic nature of fibril elongation, five replicas were studied for each temperature. Figure 1A provides a schematic of both bulk- and surface-docking pathways observed in our simulations. The peptide locked onto the fibril tip in three out of the five simulations performed at 325 K, remaining there without detaching until the end of the trajectory, i.e. for ∼1μs. In one of the three trajectories, the peptide landed on the fibril tip via the bulk-docking pathway. Snapshots from the trajectory are shown in Fig. 1B (see also Movie S1). In two of the trajectories (cf. Fig. 1C and Movie S2), the peptide followed the surface-docking pathway.

Our simulations show that nonpolar interactions are key in facilitating the surface-docking pathway. In particular, peptides interact with the nonpolar edge of the fibril by burying at least one of their nonpolar residues away from the solvent, while polar faces of the fibril remain free of peptides. Concomitantly, peptide diffusion along the fibril surface was observed to take place via the consecutive formation and rupture of nonpolar contacts between the peptide and the fibril. At the fibril tip, the peptide adopts the structure imposed by the fibril template, corresponding to an antiparallel *β*-sheet with ∼7 backbone hydrogen bonds formed between peptide and fibril. At 325 K, the latter process (independently of the docking pathway) always began with the alignment of either N- or C-terminals of the peptide with the fibril (cf. second-to-last snapshot in Fig. 1C). This process was driven by electrostatic interactions between charged side chains, i.e. by the alignment of oppositely charged residues. Subsequently, the other peptide terminal stretched out to also become aligned with the fibril, as shown in the last snapshot in Fig. 1C.

Surface-docking was also observed in simulations performed at a higher temperature (350 K; cf. Fig. S3), as well as in simulations containing shorter and longer preformed fibrils comprised of 5 and 15 peptides per *β*-sheet, respectively. We note that, when the peptide lands on the fibril surface in the vicinity of the tip, surface-docking takes place in a single step. On the other hand, in most cases docking emerges from a combination of surface and bulk diffusion processes in which the peptide approaches the tip via several binding–diffusion–unbinding events. In light of the above observations, we next present a continuum theory approach which incorporates the key processes identified in our all-atom simulations and employ it to investigate how relevant physical parameters affect the kinetics of fibril growth.

## Mesoscale model for bulk- and surface-mediated fibril growth

The kinetics of fibril growth is traditionally modeled using rate equations (15, 25–27). While such models readily yield predictions for size distributions of fibrils during aggregation in homogeneous solutions, all spatial dependence of monomer and oligomer concentrations and the effects of fibril surfaces during growth are not explicitly accounted for. Thus, a more microscopically informed model is needed to properly incorporate surface effects and quantify how these alter the overall growth kinetics of amyloid fibrils. When considering free peptides that can diffuse in the bulk and intermittently adsorb to a surface on which they can undergo further surface diffusion, the problem belongs to the category of bulk-mediated surface diffusion (BMSD) (28, 29). In the biological context, such models have been applied to study transcription factors such as the *lac* repressor, which undergoes a diffusional search along DNA to find its specific binding site (30, 31). Studies have also suggested that the ability to diffuse on the surface may enhance the rate of adsorbates in finding their target (32).

**Fig. 2.**
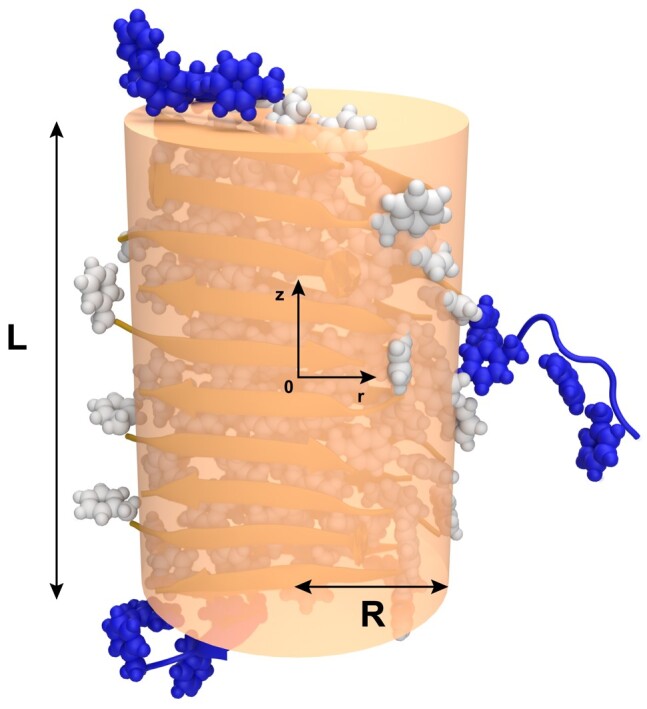
Schematic illustration of the geometry of the system. Three peptides (blue) originally in the bulk solution interact with an existing fibril (white and orange). One peptide (right) shows adsorption on fibril surface, and two other peptides are attached to both top and bottom ends of the fibril. The fibril is approximated as a cylinder (orange) to facilitate the theoretical analysis.

In our coarse-grained model, the geometry of a single fibril is approximated by a cylinder with radius *R* and length *L* as depicted in Fig. 2; we return to the merits and deficiencies of this simple approximation in the Discussion section. The center of the fibril is positioned at the origin of a cylindrical coordinate system. The fibril is immersed in a bulk solution which contains freely diffusing protein peptides quantified by the bulk density, C(r,z,t). On the curved cylindrical surface, assuming axial symmetry, the concentration of adsorbed peptides is characterized by the surface density, n(z,t). The time evolution of C(r,z,t) is governed by the diffusion equation


(1) $$\frac{\partial C(r,z,t)}{\partial t}=D_{b}\left[\frac{1}{r}\frac{\partial }{\partial r}\left(r\frac{\partial }{\partial r}\right)+\frac{\partial^{2}}{\partial z^{2}}\right]C(r,z,t),$$

where $D_{b}$ denotes the bulk diffusion coefficient. The surface density in turn is described by a 1D diffusion equation containing a flux emanating from the bulk (28):


(2) $$\frac{\partial n(z,t)}{\partial t}=D_{s}\frac{\partial^{2}n(z,t)}{\partial z^{2}}+{2\pi RD_{b}\frac{\partial C(r,z,t)}{\partial r}|}_{r=R},$$

with $D_{s}$ denoting the surface diffusion coefficient. We consider a system with a constant bulk density $C=C_{0}$ far away from the fibril such that


(3) $$\lim_{r\to \infty }C(r,z,t)=\lim_{z\to -\infty ,\infty }C(r,z,t)=C_{0}.$$

The bulk density is in turn coupled to the surface density via a reactive boundary condition (28)


(4) $$\lim_{r\to R,\;-\frac{L}{2}<z<\frac{L}{2}}C(r,z,t)=\mu n(z,t),$$

with the coupling parameter $\mu =1/(2\pi Rk_{b}\tau_{\mathrm{off}})$, where $k_{b}$ denotes the binding rate of peptides from the bulk to the surface while $\tau_{\mathrm{off}}$ is the average time for adsorbed peptides to unbind from the surface. Furthermore, at the fibril ends, to account for the interplay between diffusive transport of peptides and finite attachment kinetics, we consider mixed boundary conditions of the form


(5) $$k_{\mathrm{on}}^{b}C\mp {D_{b}\frac{\partial C}{\partial z}|}_{r<R,z=\pm \frac{L}{2}}=\;0$$

and


(6) $$k_{\mathrm{on}}^{s}n\pm {D_{s}\frac{\partial n}{\partial z}|}_{z=\pm \frac{L}{2}}=\;0,$$

with $k_{\mathrm{on}}^{b}$ and $k_{\mathrm{on}}^{s}$ denoting the attachment rates of peptides from the bulk and surface to the tips, respectively. While it has been reported that the binding rates at two ends of the fibril may not be identical (18, 33), we ignore any such asymmetries and impose reflective boundary conditions at midpoint: ${\frac{\partial C(r,z,t)}{\partial z}|}_{z=0}={\frac{\partial n(z,t)}{\partial z}|}_{z=0}=0$. Finally, with the initial conditions $C(r,z,0)=C_{0}$ and $n(z,0)=n_{0}$, we have a complete description of a system that incorporates BMSD and attachment kinetics at the fibril tips. The peptides originally in the bulk solution have two pathways to the fibril ends, one via direct binding from the bulk (i.e. bulk-docking) and another via adsorption and surface diffusion (surface-docking). To study the interplay between bulk diffusion, adsorption/desorption, surface diffusion, and fibril growth, we analytically and numerically explore the steady-state solutions of the coupled transport problem.

### Governing equations in nondimensional form

To solve Eqs. 1 and 2 with the corresponding initial and boundary conditions, the governing equations are first nondimensionalized by defining $\tilde{r}=r/R$, $\tilde{z}=z/R$, and $\tau =D_{b}t/R^{2}$. We also define $C_{0}=\mu n_{0}$ such that the dimensionless bulk and surface densities become $\tilde{C}(\tilde{r},\tilde{z},\tau )=C(r,z,t)/C_{0}$ and $\tilde{n}(\tilde{z},\tau )=n(z,t)/n_{0}$, respectively. Eqs. 1 and 2 in turn become


(7) $$\frac{\partial \tilde{C}(\tilde{r},\tilde{z},\tau )}{\partial \tau }=\left[\frac{1}{\tilde{r}}\frac{\partial }{\partial \tilde{r}}\left(\tilde{r}\frac{\partial }{\partial \tilde{r}}\right)+\frac{\partial^{2}}{\partial {\tilde{z}}^{2}}\right]\tilde{C}(\tilde{r},\tilde{z},\tau ),$$

(8) $$\frac{\partial \tilde{n}(\tilde{z},\tau )}{\partial \tau }=\frac{D_{s}}{D_{b}}\frac{\partial^{2}\tilde{n}}{\partial {\tilde{z}}^{2}}+{\frac{R}{k_{b}\tau_{\mathrm{off}}}\frac{\partial \tilde{C}}{\partial \tilde{r}}|}_{\tilde{r}=\tilde{R}}.$$

We next define two dimensionless parameters, the fibril aspect ratio ϵ=L/R and $\Delta =\frac{RD_{b}}{D_{s}k_{b}\tau_{\mathrm{off}}}$, with Δ controlling the interplay between the peptide fluxes along the surface and to/from the surface as will be discussed in more detail shortly. Now, the dimensionless radius of the fibril is scaled to $\tilde{R}=1$ such that the dimensionless fibril length $\tilde{L}=\epsilon$. The boundary conditions in turn become ${\text{lim}}_{\tilde{r}\to \infty }\tilde{C}(\tilde{r},\tilde{z},\tau )={\text{lim}}_{\tilde{z}\to -\infty ,\infty }\tilde{C}(\tilde{r},\tilde{z},\tau )=1$, ${\text{lim}}_{\tilde{r}\to 1,\;-\frac{\epsilon }{2}<\tilde{z}<\frac{\epsilon }{2}}\tilde{C}(\tilde{r},\tilde{z},\tau )=\tilde{n}(\tilde{z},\tau )$, $\tilde{C}=\pm \zeta_{b}{\frac{\partial \tilde{C}}{\partial \tilde{z}}|}_{\tilde{r}<1,\;\tilde{z}=\pm \frac{\epsilon }{2}}$, and $\tilde{n}=\mp \zeta_{s}{\frac{\partial \tilde{n}}{\partial \tilde{z}}|}_{\tilde{z}=\pm \frac{\epsilon }{2}}$, where $\zeta_{m}=\frac{D_{m}}{k_{\mathrm{on}}^{m}R}$ denote dimensionless parameters that describe the interplay between attachments kinetics and diffusive transport. Based on reported data on various protein systems that can form amyloid fibrils (27, 34–36), we estimate that $\zeta_{b}$ varies from $∼10^{-1}$ to $∼10^{6}$, thus encompassing both bulk diffusion-limited ($\zeta_{b}\ll 1$) and attachment kinetics-limited ($\zeta_{b}\gg 1$) fibril growth regimes.

Now, as briefly alluded to above, the parameter Δ is key in determining the role of the fibril surface on fibril growth kinetics. More specifically, at fixed aspect ratio (or fibril length) ϵ, in the asymptotic limit Δ→0, Eq. 8 together with the boundary conditions at the tip and fibril midpoint imply that, in steady state, $\tilde{n}=0$ along the fibril surface. In this limit, the entire fibril surface effectively acts as a sink, and hence the surface-mediated peptide flux to the fibril tips scales as L (while the bulk flux is expected to be only weaklydependent on L); consequently, dL(t)/dt≃ΓL(t)+Ω, where Γ and Ω denote constants, implying accelerated growth with L(t)≃(L(0)+Ω/Γ)exp(Γt)−Ω/Γ. On the other hand, for finite Δ, boundary layers of width $\delta ∼\Delta^{-1/2}$ are expected to develop at both fibril ends such that $\tilde{n}\to 1$ outside the boundary layers; in this limit, the surface-mediated peptide flux to the fibril tips becomes length independent such that dL(t)/dt∼ const. Therefore, at finite Δ≪1, we expect a crossover in surface-mediated fibril growth kinetics when the boundary layers become nonoverlapping corresponding to $\epsilon^{*}\Delta^{1/2}∼1$. Finally, with regard to bulk-mediated fibril growth, we expect it to be only weakly dependent on Δ via its effect on $\tilde{C}$ through the boundary condition along the fibril surface.

### Steady-state behavior I: diffusion-limited growth

**Fig. 3.**
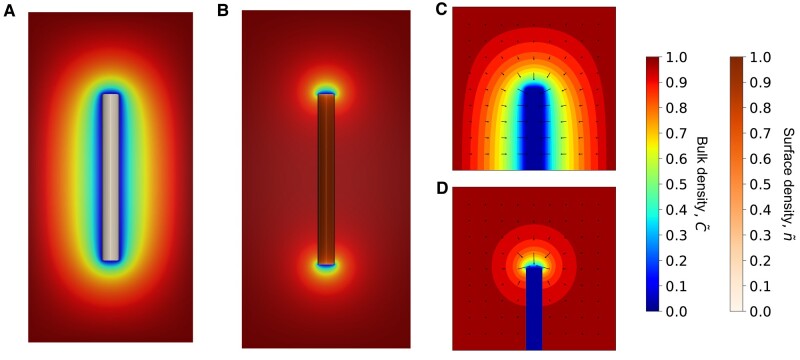
Steady-state bulk and surface peptide densities for fibril aspect ratio ϵ=20. A) 3D rendering of a slice of ${\tilde{C}}_{\mathrm{ss}}(\tilde{r},\tilde{z})$ with the cylindrical surface colored with the local magnitude of ${\tilde{n}}_{\mathrm{ss}}(\tilde{z})$ for Δ=0.001. B) 3D rendering for Δ=1,000. C) Contour plot of ${\tilde{C}}_{\mathrm{ss}}(\tilde{r},\tilde{z})$ around the fibril tip in panel A. D) Contour plot of ${\tilde{C}}_{\mathrm{ss}}(\tilde{r},\tilde{z})$ around the fibril tip in panel B. Local negative gradients in ${\tilde{C}}_{\mathrm{ss}}(\tilde{r},\tilde{z})$ are indicated using black arrows.

To verify and further elucidate the emerging physical picture of the coupled bulk- and surface-mediated fibril growth, we solve Eqs. 7 and 8 numerically for the steady-state bulk and surface densities, ${\tilde{C}}_{\mathrm{ss}}(\tilde{r},\tilde{z})$ and ${\tilde{n}}_{\mathrm{ss}}(\tilde{z})$, as discussed in the Materials and methods section. We first focus on the diffusion-limited case ($\zeta_{b}=\zeta_{s}=0$), such that any contact between a peptide and the tips leads to the immediate incorporation of the peptide to the growing fibril. This is equivalent to imposing absorbing boundary conditions at the fibril ends: $\tilde{C}(\tilde{r}<1,\tilde{z}=\pm \frac{\epsilon }{2})=\tilde{n}(\tilde{z}=\pm \frac{\epsilon }{2})=0$. Figure 3A and B shows 3D renderings of the steady-state solutions for fibrils of length ϵ=20. A vertical slice of ${\tilde{C}}_{\mathrm{ss}}(\tilde{r},\tilde{z})$ through the center of the cylinder is illustrated on the plane, while ${\tilde{n}}_{\mathrm{ss}}(\tilde{z})$ is mapped to the cylindrical surface. For Δ=0.001 (Fig. 3A), the bulk density approaches zero at the fibril surface. This case corresponds to the scenario where the entire fibril surface effectively acts as a sink for peptides. Indeed, the local gradients of the bulk density in the vicinity of the fibril tip (Fig. 3C) imply significant fluxes to both the tip and the surface. For Δ=1,000 (Fig. 3B), on the other hand, the solution conforms to the conventional view of the problem, in which the bulk density only shows variations in regions close to the fibril ends. The gradient field in Fig. 3D implies that the flux is negligible along the fibril surface, and hence the surface does not significantly contribute to the fibril growth.

**Fig. 4.**
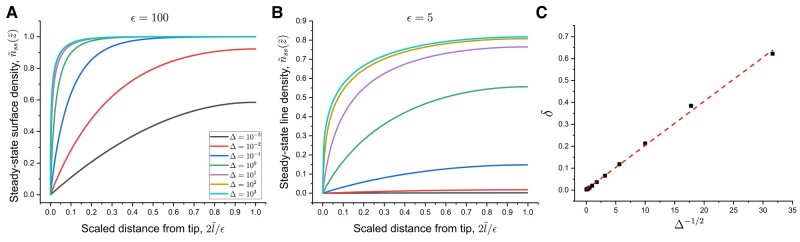
Steady-state surface densities for fibril aspect ratios ϵ=100 A) and ϵ=5 B) over a range of representative Δ values. C) Scaled boundary layer width *δ* vs. $\Delta^{-1/2}$ for ϵ=100. Red dashed line is a guide to the eye.

The effect of *Δ* on peptides along the fibril surface are further quantified by plotting the steady-state surface density profiles against the scaled distance from the tip, $2\tilde{l}/\epsilon \in [0,1]$. For a fibril with a large aspect ratio ϵ=100 (Fig. 4A), the surface density converges to 1 away from the tip for large Δ values. Also, the slope of the surface density at the tip rapidly increases at large Δ values, approaching a Heaviside step function. Indeed, the gradient of surface density at the tip shows divergent behavior as Δ increases (cf. Fig. S8). The surface density profiles have lower plateau values for shorter fibrils with ϵ=5 (Fig. 4B), indicating fewer peptides on the surface during steady-state growth. Different from longer fibrils, the surface density in this case vanishes for small Δ. As a result, for Δ<1, the boundary layer encompasses the entire fibril, while for Δ≥1, spatial variations in surface density emerge. We verify in Fig. 4C that the boundary layer width *δ*, here operationally defined as the point at which $\tilde{n}$ reaches 0.5, is indeed proportional to $\Delta^{-1/2}$ for long fibrils, consistent with our scaling argument.

**Fig. 5.**
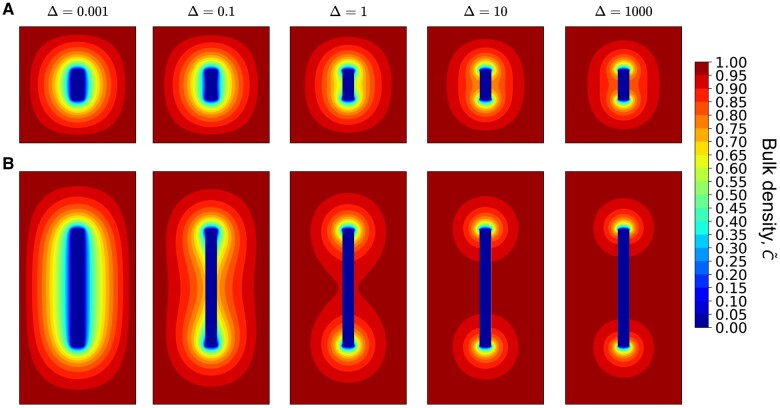
Steady-state bulk densities for two fibril aspect ratios and representative Δ values in system with diffusion-limited transport. A) ϵ=5. B) ϵ=20. We note that at low Δ values, the fibril surface as a whole acts as a sink for peptides.

Let us next discuss the combined effects of the fibril aspect ratio ϵ and Δ on steady-state bulk density profiles. In Fig. 5, we show contour plots of the steady-state bulk density for two fibrils with different aspect ratios, and compare them over a wide range of Δ values. For ϵ=5 (Fig. 5A), the bulk density at the fibril surface becomes vanishingly small for small Δ, indicating that the surface acts as an effective conduit for transporting peptides to the ends. For Δ≳1, boundary layer effects begin to emerge along the fibril surface, consistent with the transition shown in Fig. 4B. This effect is amplified for longer fibrils (ϵ=20 in Fig. 5B), for which the variations in bulk density are more sensitive to changes in Δ. The contour plot at Δ=0.001 is similar to the short fibril case, while the boundary layer starts to develop at Δ∼0.1, implying less efficient transport of peptides along the fibril surface. Further increasing Δ leads to smaller δ, and the contour lines start to converge at the two ends. An even longer fibril with ϵ=100 exhibits similar decoupling behavior at a lower value of Δ=0.001 (Fig. S9).

#### Fibril growth rate

We now turn to the implications of the above observations on the fibril growth rate. Specifically, we focus on the net amount of peptides transported to the fibril ends per unit time. Under diffusion-limited conditions considered above, the peptide flux emanating from the bulk to one end of the fibril is given by $j_{b}(r)=D_{b}{\frac{\partial C}{\partial z}|}_{z=L/2}$ such that the total amount of peptides incorporated to the growing fibril per unit time (“flow rate”) can be determined from $J_{b}=4\pi \int_{0}^{R}j_{b}\;rdr$. In steady state, the net amount of peptides that diffuse to the ends along the fibril surface equals the net amount of bulk peptides adsorbed on the surface per unit time, and thus the flow rate of peptides from fibril surface to the fibril ends, $J_{s}$, can be readily calculated via $J_{s}=2\pi R\int_{-L/2}^{L/2}j_{s}\;dz$, where $j_{s}(z)=D_{b}{\partial C/\partial r|}_{r=R}$. To illustrate how these flow rates vary in the parametric study, we focus on the dimensionless flow rates ${\tilde{J}}_{b}=J_{b}/(RD_{b}C_{0})$ and ${\tilde{J}}_{s}=J_{s}/(RD_{b}C_{0})$, as well as the total flow rate ${\tilde{J}}_{\mathrm{total}}={\tilde{J}}_{b}+{\tilde{J}}_{s}$.

**Fig. 6.**
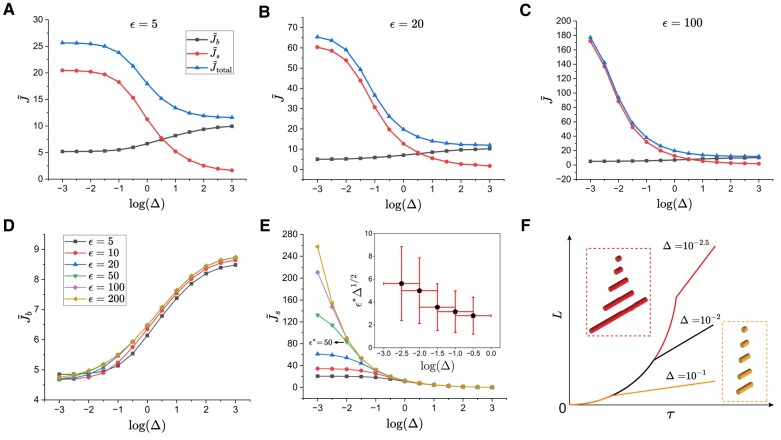
Steady-state flow rates of peptides to the fibril ends for several fibril aspect ratios ϵ and and representative Δ values under diffusion-limited transport conditions. A) ϵ=5 . B) ϵ=20. C) ϵ=100. D) Bulk flow rate. E) Surface flow rate. (Inset) Critical aspect ratio ($\epsilon^{*}$) for several Δ values. F) Schematic of fibril length vs. time with three representative Δ values. At the largest Δ, two growth kinetics is linear already at early times, while at smaller Δ, the fibril initially experiences accelerated growth prior to reaching an asymptotic linear growth regime.

The computed flow rates are shown in Fig. 6 for several representative ϵ and Δ values. For a short fibril with ϵ=5 (Fig. 6A), the trend shows that ${\tilde{J}}_{s}$ decreases with increasing Δ while ${\tilde{J}}_{b}$ increases. ${\tilde{J}}_{s}$ is the main source for the total flow rate at small Δ values, which agrees with our prior understanding that peptides on the fibril surface are instantly transported to the ends. As Δ increases, the difference between ${\tilde{J}}_{s}$ and ${\tilde{J}}_{b}$ diminishes, with ${\tilde{J}}_{b}$ eventually becoming the main contribution to the total flow rate. For longer fibrils with ϵ=20 (Fig. 6B) or ϵ=100 (Fig. 6C), ${\tilde{J}}_{s}$ is at least one order of magnitude larger than ${\tilde{J}}_{b}$ at small Δ. Interestingly, it is observed that for distinct fibril aspect ratios ϵ, ${\tilde{J}}_{b}≃{\tilde{J}}_{s}$ at $\Delta ≃\sqrt{10}$, indicating a crossover in the fibril growth kinetics from surface diffusion dominated to bulk diffusion dominated behavior. Furthermore, ${\tilde{J}}_{b}$ is only weakly dependent on ϵ as shown in Fig. 6D, while ${\tilde{J}}_{s}$ displays a much stronger dependence on both ϵ and Δ (cf. Fig. 6E). We note that for each Δ, there exists a critical aspect ratio $\epsilon^{*}$ above which the fibril experiences a constant flow rate from the surface. Given the emergence of the boundary layer $\delta ∼\Delta^{-1/2}$, we expect such a crossover to occur when $\epsilon^{*}/\delta ∼1$ or $\epsilon^{*}\Delta^{1/2}∼1$. As shown in the inset of Fig. 6E, our data is in reasonable agreement with this simple scaling argument. Finally, we note that for $\epsilon <\epsilon^{*}$, the net flow rate is proportional to fibril length, and consequently the fibril initially experiences accelerated growth (exponential in time) prior to reaching an asymptotic linear growth regime, as illustrated in the schematic in Fig. 6F.

### Steady-state behavior II: attachment rate-limited growth from bulk; diffusion-limited growth from surface

**Fig. 7.**
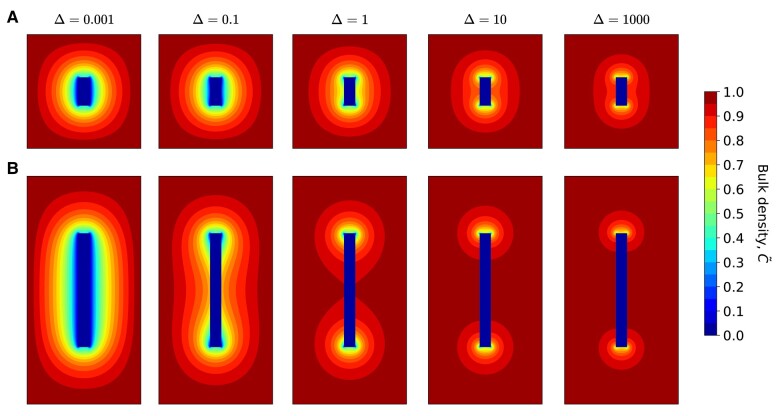
Steady-state bulk densities for two fibril aspect ratios and representative Δ values in system with attachment kinetics-limited bulk and diffusion-limited surface transport. A) ϵ=5. B) ϵ=20.

We next discuss the scenario where the incorporation of bulk peptides is attachment rate-limited ($\zeta_{b}\to \infty$), while the transport of surface peptides remains diffusion-limited ($\zeta_{s}\to 0$), corresponding to the boundary conditions ${\partial \tilde{C}/\partial \tilde{z}|}_{\tilde{r}<1,\tilde{z}=\pm \epsilon /2}=0$ and $\tilde{n}(\tilde{z}=\pm \epsilon /2)=0$. We first focus on the combined effects of the fibril aspect ratio ϵ and Δ on steady-state bulk density profiles shown in Fig. 7. At the fibril tips, there is now a finite bulk density of peptides, with a magnitude which increases with increasing Δ (cf. Fig. 7A) and asymptotically approaches one. Similar to the diffusion-limited case, the bulk density approaches zero at the fibril surface for small Δ, again indicating that the surface acts as an efficient conduit of peptides to the tips. At Δ=1, boundary layers begin to emerge affecting the bulk density for fibrils with ϵ=5. For a longer fibril (ϵ=20 in Fig. 7B and ϵ=100 in Fig. S10), similar effects emerge at Δ≲0.1, highlighting the length-dependent surface transport mechanism in steady-state fibril growth.

#### Fibril growth rate

**Fig. 8.**
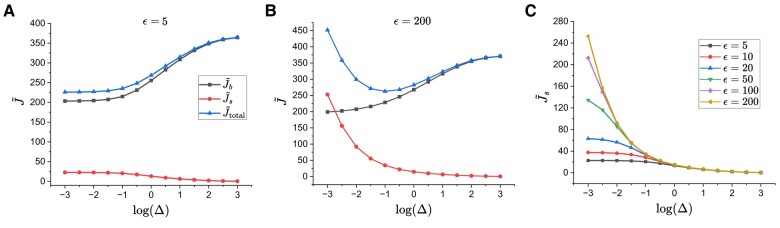
Dimensionless steady-state flow rates of peptides to the fibril ends for two fibril aspect ratios ϵ and and representative Δ values under attachment kinetics-limited bulk and diffusion-limited surface transport conditions. A) ϵ=5. B) ϵ=200. C) Surface flow rate for a range of fibril aspect ratios.

While the steady-state surface flow rate $J_{s}$ can be calculated by the same definition as in the diffusion-limited case, the bulk flow rate is now evaluated via $J_{b}=4\pi \int_{0}^{R}k_{\mathrm{on}}^{b}C(r,z=\pm L/2)rdr$, which we nondimensionalize by defining ${\tilde{J}}_{b}=J_{b}/(k_{\mathrm{on}}^{b}C_{0}R^{2})$. In Fig. 8A, we show the dimensionless flow rates for a short fibril with ϵ=5. It can be seen that while ${\tilde{J}}_{b}$ is a monotonically increasing function of Δ, ${\tilde{J}}_{s}$ is a monotonically decreasing one, resulting in a monotonically increasing net flow rate. For a much longer fibril (ϵ=200 in Fig. 8B), on the other hand, the significantly more rapid variation of ${\tilde{J}}_{s}$ with Δ results in a net flow rate with a minimum at a specific Δ. Finally, we note that ${\tilde{J}}_{s}$ in Fig. 8C shares the same behavior as in the bulk diffusion-limited case (Fig. 6E), as the governing equation and boundary conditions for surface peptides remain unchanged.

### Steady-state behavior III: attachment rate-limited growth from bulk and surface

As a final representative scenario, let us consider the case for which the fibril growth is attachment rate-limited for peptides emanating from both the bulk and the surface. In this case, the bulk density is always at $C_{0}$, while the surface density equals $C_{0}/\mu$ in steady state. Hence, the total flow rate to the tips can be readily obtained as $J_{\mathrm{total}}=k_{\mathrm{eff}}C_{0}$, where $k_{\mathrm{eff}}=2\pi R^{2}k_{\mathrm{on}}^{b}+2k_{\mathrm{on}}^{s}/\mu$. We note that $J_{\mathrm{total}}$ is independent of fibril length.

## Estimation of key parameters from MD simulations

**Fig. 9.**
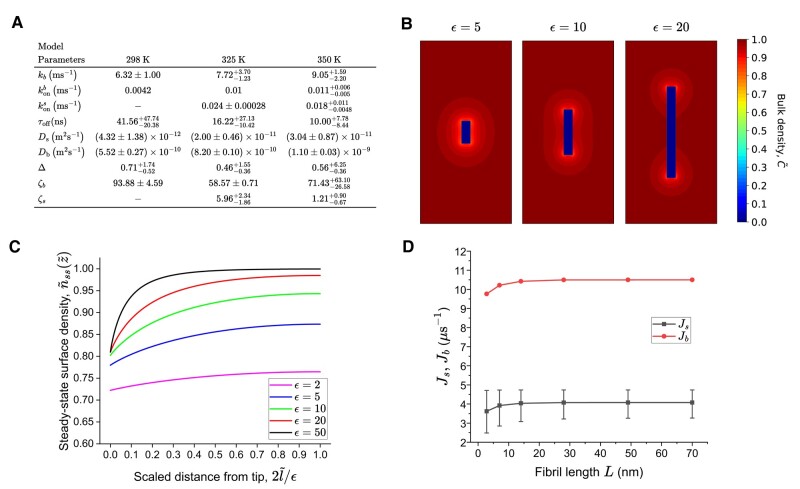
A) Computed physical quantities from MD simulations for fibrils with aspect ratios ϵ=3.86 at 298, 325, and 350 K, from which we estimate the key dimensionless parameters Δ, $\zeta_{b}$, and $\zeta_{s}$. B) Corresponding steady-state bulk densities from the coarse-grained model at 325 K with $\zeta_{b}\to \infty$, $\zeta_{s}=5.96$, and Δ=0.46 for aspect ratios ϵ=5 (left), ϵ=10 (middle), and ϵ=20 (right). C) Steady-state surface densities for fibrils with ϵ=2, 5, 10, 20, and 50. D) Predicted steady-state bulk and surface flow rates vs. fibril length.

To further elucidate the emerging picture of the fibril growth kinetics, the relevant physical quantities used in the continuum model were estimated from MD trajectories to identify the dominant growth mechanism. More specifically, simulations were performed for systems which contained preformed fibrils with three aspect ratios, namely ϵ=1.79, 3.86, and 5.29. We also systematically studied how temperature affects fibril growth by collecting data at 298, 325, and 350 K, respectively. The results for the intermediate length fibril with ϵ=3.86 are tabulated in Fig. 9A. (The procedures for estimating the various physical quantities are detailed in Supplementary material.) We note that $D_{b}$, $D_{s}$, $k_{b}$, $k_{\mathrm{on}}^{b}$, and $k_{\mathrm{on}}^{s}$ increase with temperature, while $\tau_{\mathrm{off}}$ decreases. These rather expected dependencies on temperature, however, do not lead to monotonically increasing or decreasing temperature dependencies for Δ, $\zeta_{b}$, or $\zeta_{s}$.

More insights into this nonmonotonic behavior can be inferred from an analysis of the state of the peptide in MD simulations. In particular, for the intermediate length fibril (ϵ=3.86), the percentage of trajectories in which the peptide irreversibly binds to one of the fibril tips decreases from 100% at 350 K to 60% at 325 K. At 298 K, only one out of the five trajectories ends up with the peptide incorporated into the tip. This temperature-dependent trend is observed for fibrils of all lengths. In the other trajectories, the peptide intermittently binds and unbinds from the fibril surface while spending more time on the surface as temperature decreases. More quantitatively, given that the steady-state surface density for long fibrils is given by $n_{0}∼\mu^{-1}C_{0}$, where $\mu^{-1}=2\pi Rk_{b}\tau_{\mathrm{off}}$, we readily estimate that $n_{0}^{298\;K}/n_{0}^{350\;K}∼2.9$ at constant bulk concentration. This enhancement in surface density is consistent with experimental data on the temperature dependence of secondary nucleation (37), which is observed more frequently at low temperatures.

The interplay between attachment kinetics and diffusive transport in this specific system is reflected in the estimated values of $\zeta_{b}$ and $\zeta_{s}$. At 325 K, the estimated values $\zeta_{s}\approx 6$ and $\zeta_{b}\approx 58\gg 1$ suggest that the fibril growth from the bulk is attachment rate-limited, while the surface-mediated growth is neither purely attachment rate nor diffusion-limited. We have thus numerically obtained the steady-state solutions of the continuum model corresponding to the parameter values $\zeta_{b}\to \infty$ and $\zeta_{s}=5.96$. Using Δ=0.46 from the table in Fig. 9A, the contour plots of the steady-state bulk densities for fibrils with ϵ=5, 10, and 20 are shown in Fig. 9B. First, it is observed that the variations in the bulk density are smaller than those in the diffusion-limited case. Second, fibril tips act independently for fibrils with aspect ratio ϵ≳20, corresponding to fibril length ∼15 nm. This observation is further confirmed upon inspection of the surface density profiles shown in Fig. 9C. Finally, when plotting the predicted dimensional bulk and surface flow rates, $J_{b}=k_{\mathrm{on}}^{b}C_{0}R^{2}{\tilde{J}}_{b}$ and $J_{s}=RD_{b}C_{0}{\tilde{J}}_{s}$ in Fig. 9D, we observe that $J_{b}≳2J_{s}$ over a wide range of fibril lengths. This implies that bulk peptide transport contributes more to the fibril growth than the surface one in this particular system with the specific set of physical parameters. Moreover, both flow rates are monotonically increasing functions of fibril length and become constant for L≳15 nm.

Visual analysis of our MD simulations performed using short (ϵ=1.79) and long (ϵ=5.29) fibrils reveals a trend that is consistent with the predictions from continuum theory. Specifically, in the case of short fibrils, the peptide is observed to spend only a small amount of time on the fibril surface before either unbinding or locking onto the tip, leading to a low surface density as predicted from the continuum theory (cf. the data for ϵ=2 in Fig. 9C and Movie S3). In contrast, for long fibrils, the peptides display several binding–unbinding events before locking onto the tip, thereby effectively increasing the surface density as predicted (cf. the data for ϵ=5 in Fig. 9C and Movie S4). Finally, we note that the higher surface densities associated with long fibrils are conducive to the formation of new oligomers via secondary nucleation (cf. Fig. S12 and Movie S5).

## Discussion

Amyloid fibrils, the insoluble and mechanically stable *β*-sheet structures, are associated with the pathology of many fatal diseases. Despite the progress made in determining the molecular structures of the fibrils, the biophysics of nucleation, growth, and proliferation thereof remain poorly understood at the molecular level. To address this knowledge gap, in this work, we combined unbiased, all-atom MD simulations with a continuum theory. Intriguingly, in addition to the commonly invoked dock-and-lock growth mechanism from bulk solution, our MD simulations revealed that peptides frequently interact with the fibril surface and bind to fibril tips via surface diffusion, suggesting that a *surface-docking* process may play an important role in the overall fibril growth kinetics. Furthermore, our MD results are consistent with previous experimental studies showing that fibril elongation increases at higher temperatures, whereas secondary nucleation decreases at higher temperatures (9, 38). Experimental studies also have shown that perturbing the binding of the peptide to the fibril surface can be a pathway to inhibit secondary nucleation (9, 39).

Motivated by these observations, a continuum model was formulated to facilitate the analysis of this multiscale problem. The model accounts for diffusive transport of peptides both in the bulk solution and along the fibril surface, binding and unbinding of peptides from the fibril surface, and attachment kinetics of peptides incorporated into the fibril tips from the bulk solution and the surface. The physical parameters that affect the fibril growth were expressed in terms of four dimensionless parameters, ϵ, Δ, $\zeta_{b}$, and $\zeta_{s}$, which collectively control the steady-state behavior of a growing fibril. The effects of the relevant physical parameters on the growth kinetics were investigated and distinct growth regimes were identified. We also demonstrated how the relevant model parameters can be estimated from simulated MD trajectories.

From the theory, several growth regimes were identified, corresponding to diffusion- or attachment kinetics-limited behavior in the bulk solution and/or the fibril surface. The key observations can be summarized as follows. First, in all regimes, the fibril growth rates are proportional to the bulk peptide concentration. Second, under diffusion-limited conditions for the peptides on the fibril surface, an accelerated growth regime emerges for sufficiently strong peptide-fibril surface interactions. In this regime, the fibril grows exponentially in time: L(t)≃(L(0)+Ω/Γ)exp(Γt)−Ω/Γ, where Γ and Ω denote constants. This growth phase persists until the fibril reaches a characteristic length $L^{*}∼R\Delta^{-1/2}=(D_{s}Rk_{b}\tau_{\mathrm{off}}/D_{b}{)}^{1/2}$, beyond which the fibril enters a linear growth regime with $dL(t)/dt≃f(L^{*})$. Third, under attachment kinetics-limited conditions for growth from the bulk and the fibril surface, fibrils exhibit only linear growth in time with a velocity $V=k_{\mathrm{eff}}C_{0}$, where the effective rate constant $k_{\mathrm{eff}}\propto (2\pi R^{2}k_{\mathrm{on}}^{b}+2k_{\mathrm{on}}^{s}/\mu )$ has contributions from both bulk-docking and surface-docking.

We envision several extensions of the work reported in this manuscript. First, it would be very interesting—but admittedly computationally very challenging—to study peptide-fibril interactions at the molecular scale and extract the relevant dimensionless parameters over a wide range of amino acid sequences and peptide lengths. Second, we note that more accurate geometrical approximations of a single proto-fibril can be employed in the mesoscale model, which currently overestimates the surface area available for binding. For example, the tips may be approximated by rectangles/ellipses, and the corresponding binding surfaces by rectangles/segments of a curved cylindrical surface, such that the overall geometry can be viewed as a cuboid or an elliptic cylinder with reduced available surface area for peptide binding. While such modifications will lead to some quantitative changes in the reported results, we do not expect the qualitative results to differ from the current analysis. Third, the continuum model can be readily extended to account for the formation kinetics of higher order structures, i.e. fibril bundles of two or more proto-fibrils with a helical motif (40, 41); the cylindrical approximation, in fact, becomes more accurate with such morphologies.

We would be remiss without recognizing that translating our results to the crowded, heterogeneous cellular milieu is a very challenging task indeed. In living cells and organisms, the formation of amyloid fibrils takes place over macroscopic time scales under nonequilibrium conditions while interacting with other entities such as lipid membranes (42–44) and biomolecular condensates (45–47). In the former case, the presence of membranes may cause uneven peptide distributions, affecting both bulk/surface diffusion and attachment rates. In the latter case, evidence has shown that these protein-rich condensates may provide necessary conditions for amyloid fibrils to nucleate and grow (48, 49). The proliferation of fibrils may then lead to the consumption of the condensates and formation of higher order structures that resemble the neurofibrillary tangles found in many neurodegenerative diseases (50, 51). A more fundamental understanding of such interactions and processes, however, remains elusive.

## Conclusion

This study has introduced a quantitative framework for assessing and interpreting the growth of amyloid-like fibrils emlpoying a closely integrated atomistic MD simulation and mesoscale continuum theory approach. The MD simulations provide indispensable insights into the governing processes at the molecular scale, while the continuum model, parameterized by the MD trajectories, enables a quantitative study of fibril growth kinetics across mesoscopic length and time scales. More specifically, our work has shown that both bulk and surface diffusion, as well as attachment kinetics, are key factors in controlling the growth of amyloid-like fibrils. Our hope is that a better quantitative understanding of such processes may facilitate the identification of optimized routes between bulk and surface pathways with the goal of either increasing/decreasing fibril growth rate or inhibiting secondary nucleation in various amyloid systems for treatment purposes.

## Materials and methods

### System design and MD simulations

To investigate the kinetic of the of fibril growth, extensive simulations were performed at three temperatures (298, 325, and 350 K). The systems studied here were prepared in two steps. First, a preformed fibril consisting of 10, 20, or 30 peptides was placed in a cubic box of 10.1×10.1×10.1 nm ^3^. Second, a peptide with the same sequence, i.e. Ac-(FKFE) _2_-NH _2_, was inserted randomly in the box with a minimum distance greater than 2 nm from the fibril. The simulation box was then solvated and the total energy of the system was minimized using the steepest descent method. Subsequently, a 4-ns equilibration in the NVT ensemble with a position restraint on the peptides was performed to relax the water molecules. We note that this choice of equilibration time is significantly longer than those (∼0.1 ns) typically employed in simulations (52–55). All production runs were carried out in the NPT ensemble with periodic boundary conditions and simulated times ranging from 0.5 to 4 μs.

### Software and parameters

Recently, the ability of the peptides to populate the fibril tip via both bulk and surface pathways was observed with both CHARMM36m and Amber99sb-ILDN force fields, suggesting that this behavior is generic (24). In the present work, the Amber99sb-ILDN force field combined with the TIP3P model for water (56, 57) was employed to quantify these pathways. All simulations were carried out using GROMACS version 2020 (58). The leapfrog algorithm was employed to integrate the equations of motion with a 2-fs time step (59). The Parrinello–Rahman barostat ($\tau_{p}=2.0$ ps) was employed to maintain the pressure of the system at 1 bar (60). Temperature was controlled by coupling the peptides and the solvent separately to a velocity-rescale thermostat ($\tau_{t}=0.1$ ps) (61). The cutoff for short range van der Waals and electrostatic interactions was set to 1.0 nm. The smooth Particle Mesh Ewald algorithm (62) was employed in turn to compute the long-range electrostatic interactions. VMD was used for structure visualization (63).

### Numerical method for solving steady-state equations

The nondimensionalized equations (Eqs. 7 and 8) were solved numerically using the forward in time, centered in space (FTCS) scheme on a 2D uniform grid with spacing $d\tilde{r}=d\tilde{z}=0.01$. $\tilde{C}(\tilde{r},\tilde{z},\tau )$ was obtained for $\tilde{z}>\epsilon /2$ and $\tilde{r}>1$; $\tilde{n}(\tilde{z},\tau )$ was obtained for $\tilde{r}=1$ and $0<\tilde{z}<\epsilon /2$. The full peptide bulk density profiles were constructed by mirroring with respect to both $\tilde{r}=0$ and $\tilde{z}=0$. The first and second derivatives were both evaluated to second-order accuracy to minimize errors in fluxes along the fibril surface. We also note that due to limits on the numerical grid size, extreme values of Δ>1,000 and Δ<0.001 might not retain the same accuracy as the others. To reduce the boundary effects for different fibril aspect ratios, the grid size adapts with different aspect ratios: 1,000×1,000 for ϵ=5 and 1,000×10,000 for ϵ=100. A sufficiently small time step $dt=2\times 10^{-5}$ was chosen to ensure numerical stability for the FTCS scheme. The steady-state solutions in the parametric study with ϵ<200 were obtained in less than $2\times 10^{8}$ time steps, while the case with ϵ=200 reached steady state in $4\times 10^{8}$ time steps.

## Supplementary Material

pgae045_Supplementary_Data

## Data Availability

All data that support the findings of this study are available in the manuscript and the Supplementary material.
