# Supplementary material for: Growth kinetics of amyloid-like fibrils: An integrated atomistic simulation and continuum theory approach
Source: PNAS Nexus. 2024 Feb 1;3(2):pgae045. doi: 10.1093/pnasnexus/pgae045 (PMC11079572; doi:10.1093/pnasnexus/pgae045)
Supplement: pgae045_Supplementary_Data [file pgae045_supplementary_data.zip › PNASNEXUS-PNASNEXUS-2023-00893R-s06.pdf]

Supporting Information for

Growth kinetics of amyloid-like fibrils: An integrated atomistic simulation and continuum theory approach

Ruoyao Zhang, Sharareh Jalali, Cristiano Luis Dias and Mikko P. Haataja

Corresponding author: Mikko P. Haataja
E-mail: mhaataja@princeton.edu

This PDF file includes:

Supporting text
Figs. S1 to S12
Tables S1 to S2
Legends for Movies S1 to S5
SI References

Other supporting materials for this manuscript include the following:

Movies S1 to S5

Supporting Information Text

Simulation setup

All-atom molecular dynamics simulations in explicit solvent were performed in $10.1 \times 10.1 \times 10.1$ nm^3 cubic boxes containing a pre-formed fibril and a peptide. The pre-formed fibril is made by packing non-polar faces of two antiparallel β-sheets against each other. The peptide is inserted randomly within the simulation box at a distance > 2 nm from the fibril. The peptide sequence consists of a strictly alternating pattern of non-polar (phenylalanine F) and polar (glutamic acid E, and lysine K) amino acids, i.e., Ac-(FKFE)$_2$-NH$_2$ – see Fig. S1.

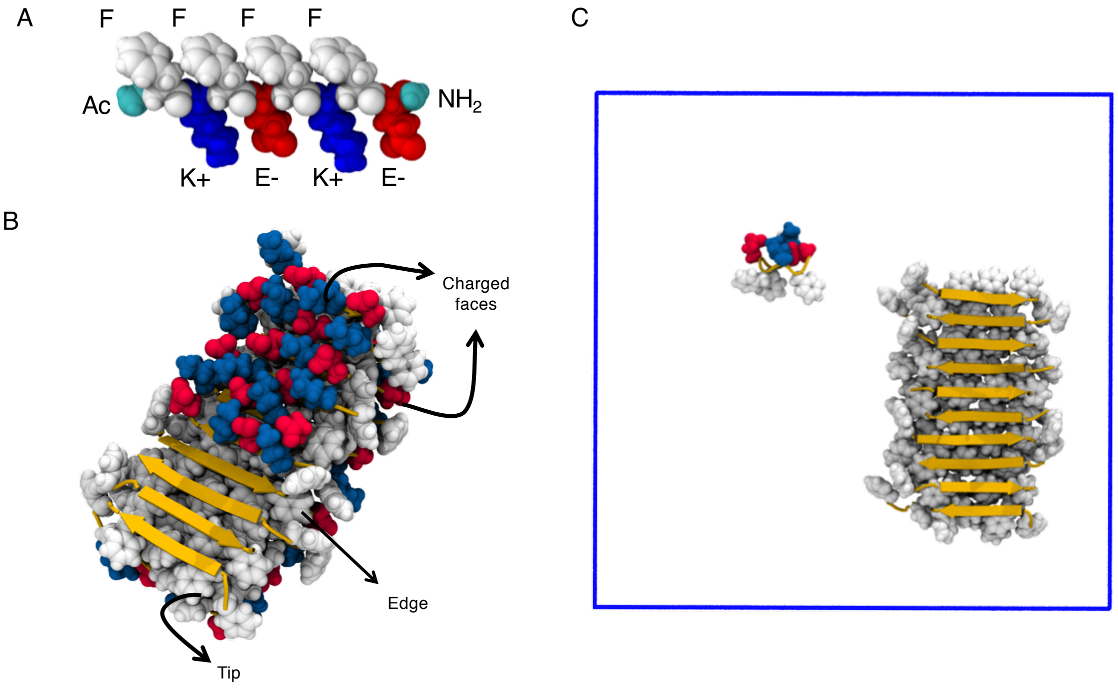

Fig. S1. (*A*) Schematic representation of the atomic structure of the strictly alternating amphipathic Ac-(FKFE)$_2$-NH$_2$ peptide. (*B*) Cross-β structure made of two laminated β-sheets with a dry core and two solvent exposed polar faces. White, red and blue colors are used to indicate phenylalanine (F), glutamic acid (E) and lysine (K), respectively. (*C*) Simulation box containing a pre-formed fibril and a peptide. Water molecules are omitted from the visualization for clarity.

To investigate different scenarios of fibril growth, simulations were performed at three temperatures (i.e., 298 K, 325 K, and 350 K) and using fibrils with different aspect ratios. Short, medium, and long fibrils are made with β-sheets containing 5, 10, and 15 peptides, corresponding to fibril aspect ratios $\epsilon = 1.79$, 3.86 and 5.29, respectively. For each temperature and fibril length, simulations were repeated at least five times due to stochastic nature of the dock-and-lock mechanism. Table S1 provides a summary of the simulations carried out in this study.

Table S1. Number of simulations performed at different temperatures vs. fibrils aspect ratios $\epsilon = L/R$. The approximate lengths of the simulated trajectories are reported in microseconds.

ϵ \ Temp.	298 K	325 K	350 K
1.79	—	5×0.5 μs	5×0.5 μs
3.86	5×4 μs	5×1.5 μs	7×1.5 μs
5.29	—	5×1 μs	5×1 μs

Determining the state of the peptide

The peptide in the simulation box is either solvated, or bound to the fibril at its surface or tip. To distinguish between solvated and fibril-bound states, the minimum distance between pairs of peptide-fibril atoms is computed for each trajectory. This quantity is shown in red in Fig. S2. A cut-off distance of 0.3 nm is used to determine if the peptide is bound to the fibril or solvated. To distinguish between edge- and tip-bound states, the minimal distance between center-of-mass (COM) of fibril and atoms of the peptide is also computed (shown in blue in Fig. S2). Whenever the peptide is bound to the medium-length fibril

with a COM-distance greater than 2 nm (see dashed lines), it is found to be bound to its tip. For all other COM-distances in which the peptide is bound to the fibril, it is defined as surface-bound.

The two distances shown in Fig. S2 allow us to determine the state of the peptide at any given time. Accordingly, we find that one, three, and all seven trajectories simulated at 298 K, 325 K, and 350 K, respectively, end up with the peptide locked onto the fibril – see Fig. S2. Note that trajectories in which the peptide does not lock onto the tip were extended to more than 2.5 μs. Minimal and COM distances also allow us to determine the path taken by the peptide to reach the tip. For example, in the first trajectory at 325 K, the peptide lands on the fibril surface at ~750 ns (i.e., its COM distance is smaller than 2 nm). This is followed by the diffusion of the peptide (during which it does not detach from the fibril surface) until it reaches the fibril tip via the *surface-docking* pathway. The last trajectory at 325 K in turn provides an example of the *bulk-docking* pathway.

Distances for short and long fibrils are depicted in Figs. S3 and S4. Table S2 summarizes the number of trajectories in which the peptide locks onto the tip via the bulk- and surface-docking pathways. This table notably shows that, with the exception of 298 K, surface-docking is essentially as an important pathway as bulk-docking.

Table S2. Number of trajectories in which the peptide followed bulk- and surface-docking pathways before locking onto the tip. The total number of trajectories simulated for each condition is shown in Table S1.

Temp. / ϵ	298 K		325 K		350 K	
	Bulk-	Surface-	Bulk-	Surface-	Bulk-	Surface-
1.79	—	—	3	2	2	2
3.86	1	0	1	2	4	3
5.29	—	—	2	0	3	2

The average time between consecutive binding and unbinding events to the fibril surface accounts for τ_{off}, i.e., the residence time. The average value of τ_{off} was evaluated over each trajectory. Finally, the average values of multiple trajectories and the corresponding standard deviations were reported at each temperature.

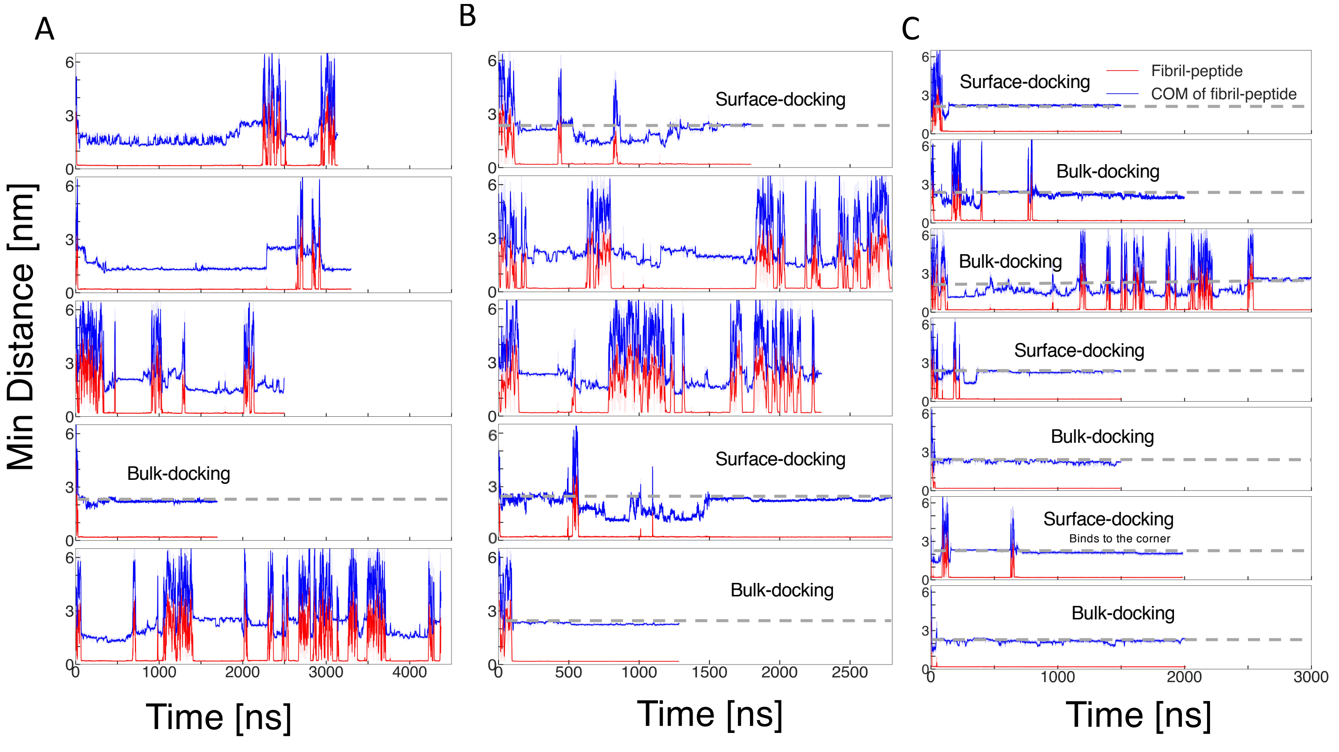

Fig. S2. Simulations performed with a medium-length fibril with aspect ratio $\epsilon = 3.86$. Minimal distances between peptide-fibril atoms (in blue) and those between COM of the fibril and atoms comprising the peptide (in red) computed at (*A*) 298 K, (*B*) 325 K and (*C*) 350 K. Dashed lines indicate tip-bound configurations.

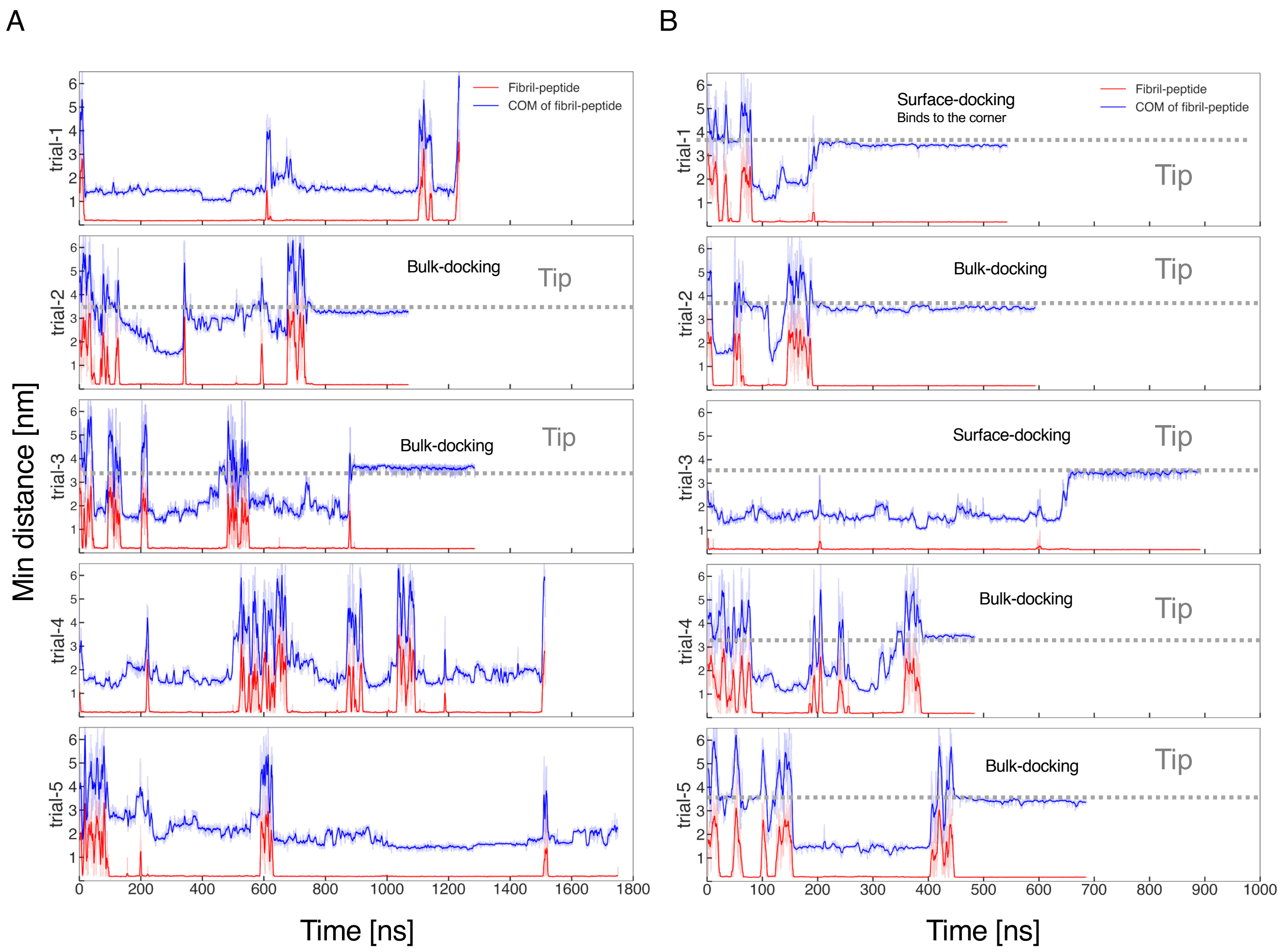

Fig. S3. Simulations performed with a long fibril with aspect ratio $\epsilon = 5.29$. Minimal distances between peptide-fibril atoms (in blue) and those between COM of the fibril and atoms comprising the peptide (in red) computed at (*A*) 325 K and (*B*) 350 K. Dashed lines indicate tip-bound configurations.

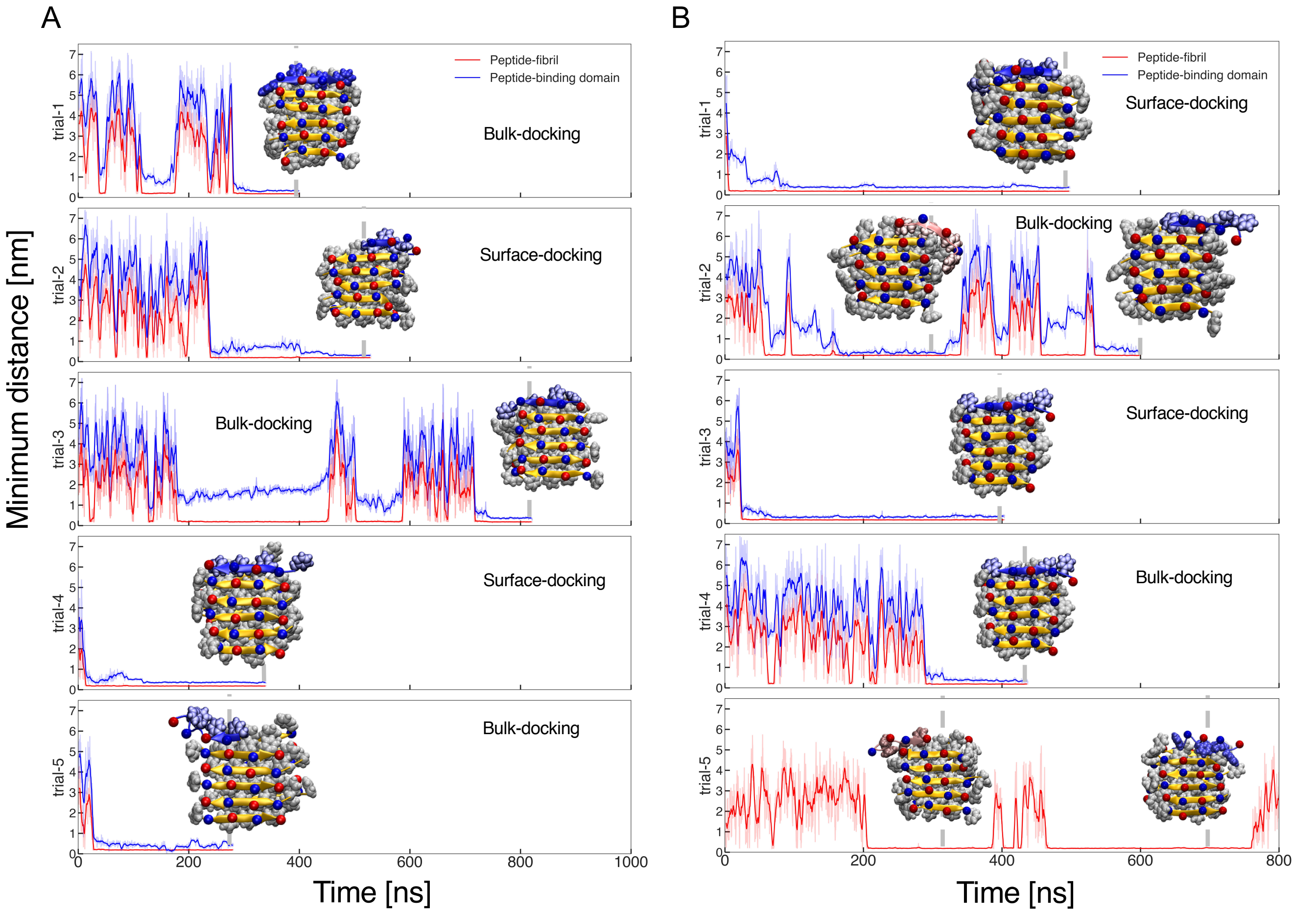

Fig. S4. Simulations performed with a short fibril with aspect ratio $\epsilon = 1.79$. Minimal distances between peptide-fibril atoms (in red) and those between the peptide and the targeted tip, i.e., final binding spot (in blue) computed at (*A*) 325 K and (*B*) 350 K. The locked conformation of the peptide with lysine and glutamic acids represented by blue and red spheres, respectively, are shown.

Diffusion coefficient computed from MD simulations

The diffusion coefficient D_b is estimated in our simulations from the mean square displacement (MSD) of the peptide's COM via Einstein's relation

$$D_b = \frac{1}{2dt} \lim_{t\to\infty} \langle (\vec{r}(t) - \vec{r}(0))^2 \rangle,$$

where d denotes the dimensionality of the system, and $\vec{r}(t)$ corresponds to the position of the center-of-mass (COM) of the peptide at time t. Independent segments of our trajectories in which the peptide is adsorbed onto the fibril surface are used to compute D_s. To obtain D_b, three additional simulations were performed in large solvated boxes without the presence of a fibril. Figure S5 shows the MSD of the peptide in simulations performed with medium-length fibril at 298 K, 325 K and 350 K. The average MSD is shown in red while the black dashed line corresponds to the line of best fit of this average (1–3). Figure S6 in turn shows D_s computed for the long fibril at 325 K and 350 K. The computed diffusion coefficients from our simulations are listed in Fig. 9A of the main text.

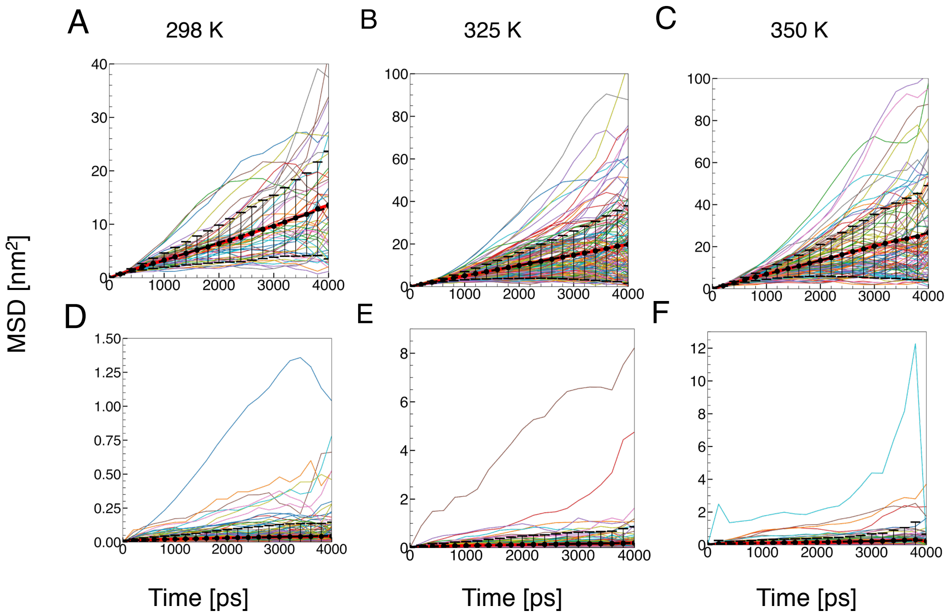

Fig. S5. Mean squared displacement (MSD) of the peptide's COM in bulk solution at (*A*) 298 K, (*B*) 325 K and (*C*) 350 K. The MSD of the peptide's COM as it diffuses on the fibril surface is shown at (*D*) 298 K, (*E*) 325 K and (*F*) 350 K. The average MSD is shown in red with error bars representing the standard deviation. Linear fits of the average MSD are shown using black dashed lines.

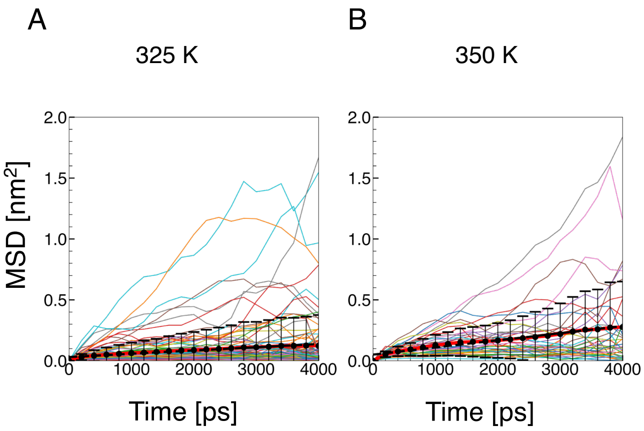

Fig. S6. Mean squared displacement of the COM of the peptide on the fibril surface for the long fibril at (*A*) 325 K and (*B*) 350 K. The average MSD is shown in red with error bars representing the standard deviation. Linear fits of the average MSD are shown using black dashed lines.

Estimation of rate constants from MD trajectories

The rate with which the peptide locks onto the tip from bulk is defined as

$$k^b_{\text{on}} = \frac{V_b}{A_{\text{tip}} t_b},$$

where V_b corresponds to the relevant bulk volume around the tip shown by hemispheres in Fig. S7*A*, i.e., $\frac{4}{3}\pi R_b^3$, A_{tip} is the area of both tips, i.e., $2\pi R^2$, and t_b is the total time the COM of the peptide spends in the two hemispheres before locking permanently onto the tip by forming at least four hydrogen bonds. A capture radius $R_b = 2$ nm is used to compute the relevant bulk volume. In the same vein, the rate with which the peptide locks onto the tip from the surface is defined as

$$k^s_{\text{on}} = \frac{l_b}{t_s},$$

where $l_b = R_b = 2$ nm denotes the capture length (see Fig. S7*A*), while t_s is the total time the peptide spends within the capture region. In order to compute t_s, all the frames in which the minimal atomic distance between the peptide and the fibril is less than 0.3 nm, i.e., bound state, and z coordinate of the COM of the peptide is between Z_1 and Z_2 are counted and multiplied by time intervals between two frames.

All trajectories in which the peptide locks onto tip from the bulk and the surface were used to compute k^b_{on} and k^s_{on} values, respectively. For the medium-length fibril, this includes seven trajectories at 350 K, three at 325 K, and one at 298 K – see Table S1.

The binding rate of the peptide to the fibril surface (k_b) is computed by counting the number of binding events to the surface divided by the time the peptide spends close to the fibril (i.e., the cylinder surrounding the fibril in Fig. S7*B*) normalized by the area of the fibril edges (i.e., $2 \times 2l_b \times$ thickness), and the peptide concentration in the captured bulk volume, i.e., $\left(\frac{1}{V_{\text{cylinder}} - V_{\text{fibril}}}\right) \text{nm}^{-3}$.

Estimates of k_b computed from our trajectories at 298 K, 325 K, and 350 K are provided in Fig. 7*A* of the main text.

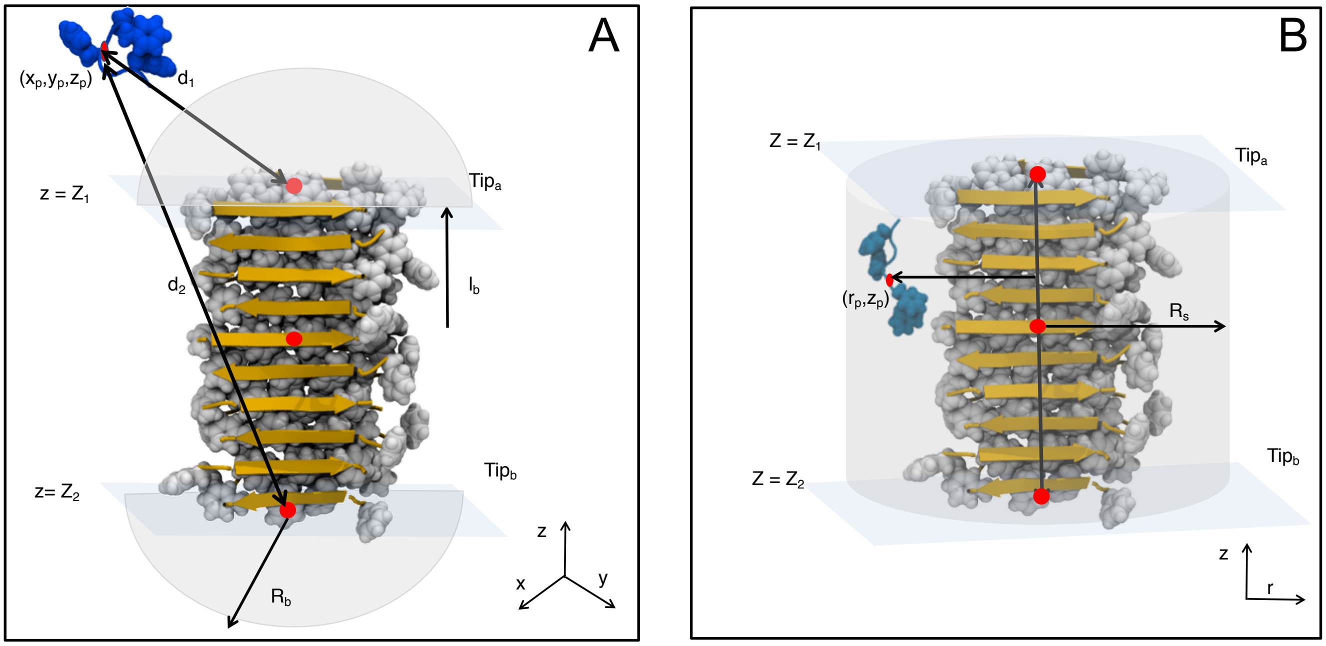

Fig. S7. (*A*) Schematic representation of a fibril, a peptide and the "capture hemispheres" (of radius R_b=2) encompassing both tips. Peptide residing within one of the hemispheres is incorporated into the tips via bulk-docking as quantified by the attachment rate k^b_{on}. For surface docking, a peptide residing on the surface within the capture length l_b is incorporated into the tips as quantified by the attachment rate k^s_{on}. (*B*) Schematic representation of a fibril, a peptide and a cylindrical capture volume around the fibril surface. A peptide residing within the capture volume is adsorbed onto the fibril surface as quantified by the binding rate k_b.

Estimation of residence time from MD trajectories

The average time between consecutive binding and unbinding events to the fibril surface accounts for τ_{off}, i.e., the residence time. The average value of τ_{off} over all trajectories is reported in Fig. 9*A* of the main text.

Secondary nucleation

We performed six simulations with six peptides and pre-formed medium-length fibril at 350 K. In two of the trajectories, the peptides aggregate and form a new nucleus on the surface of the parent fibril.

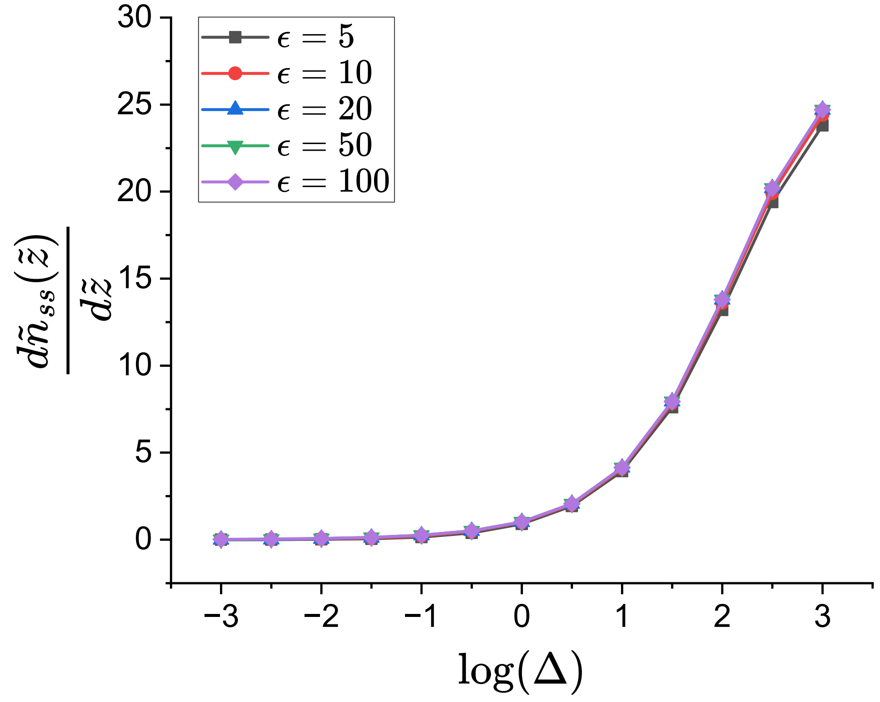

Fig. S8. Gradient of steady-state surface density at the tip for various values of ϵ and Δ.

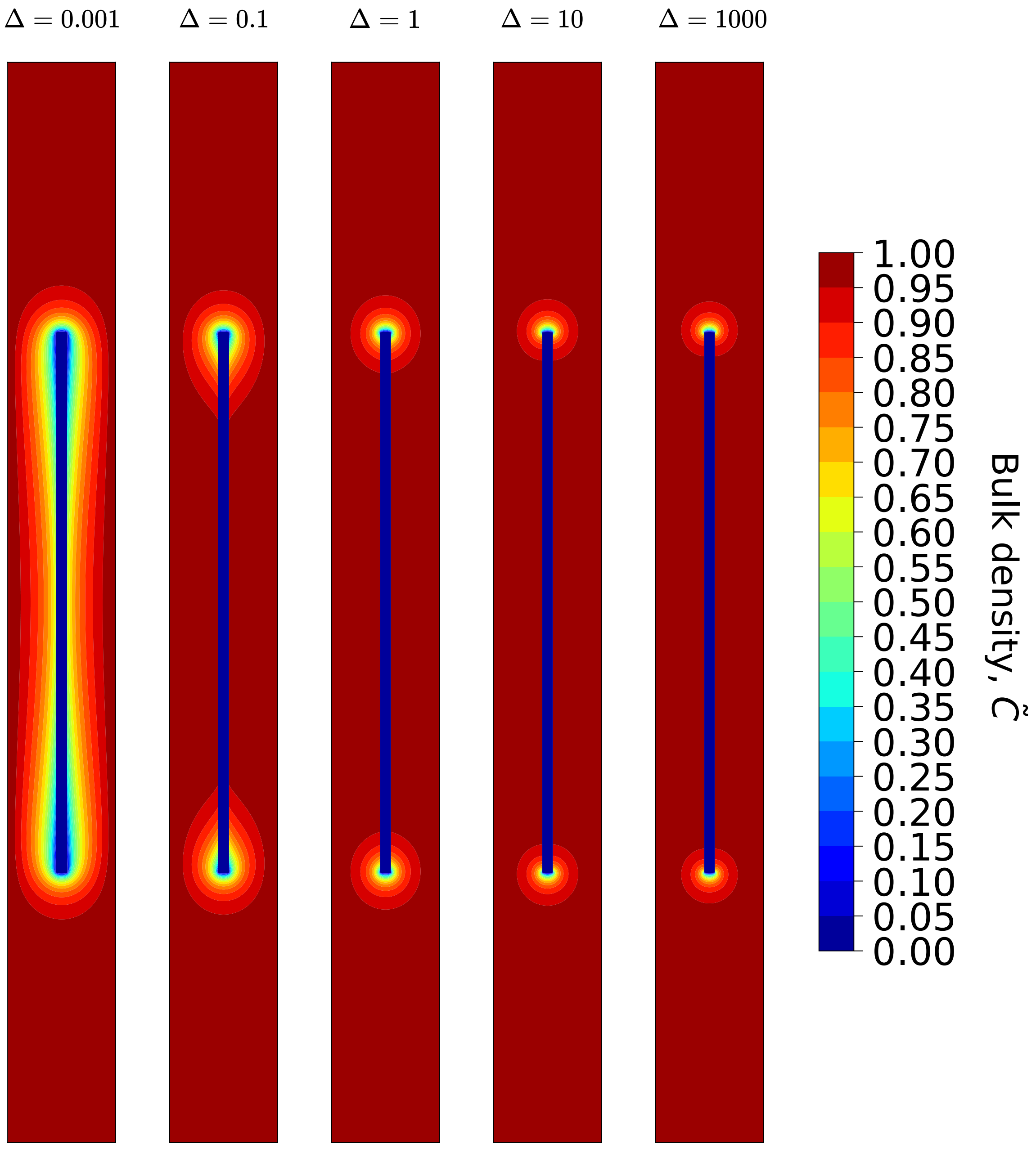

Fig. S9. Diffusion-limited growth: steady-state bulk density profiles ($\tilde{C}$) for a fibril with aspect ratio $\epsilon = 100$ and various Δ values.

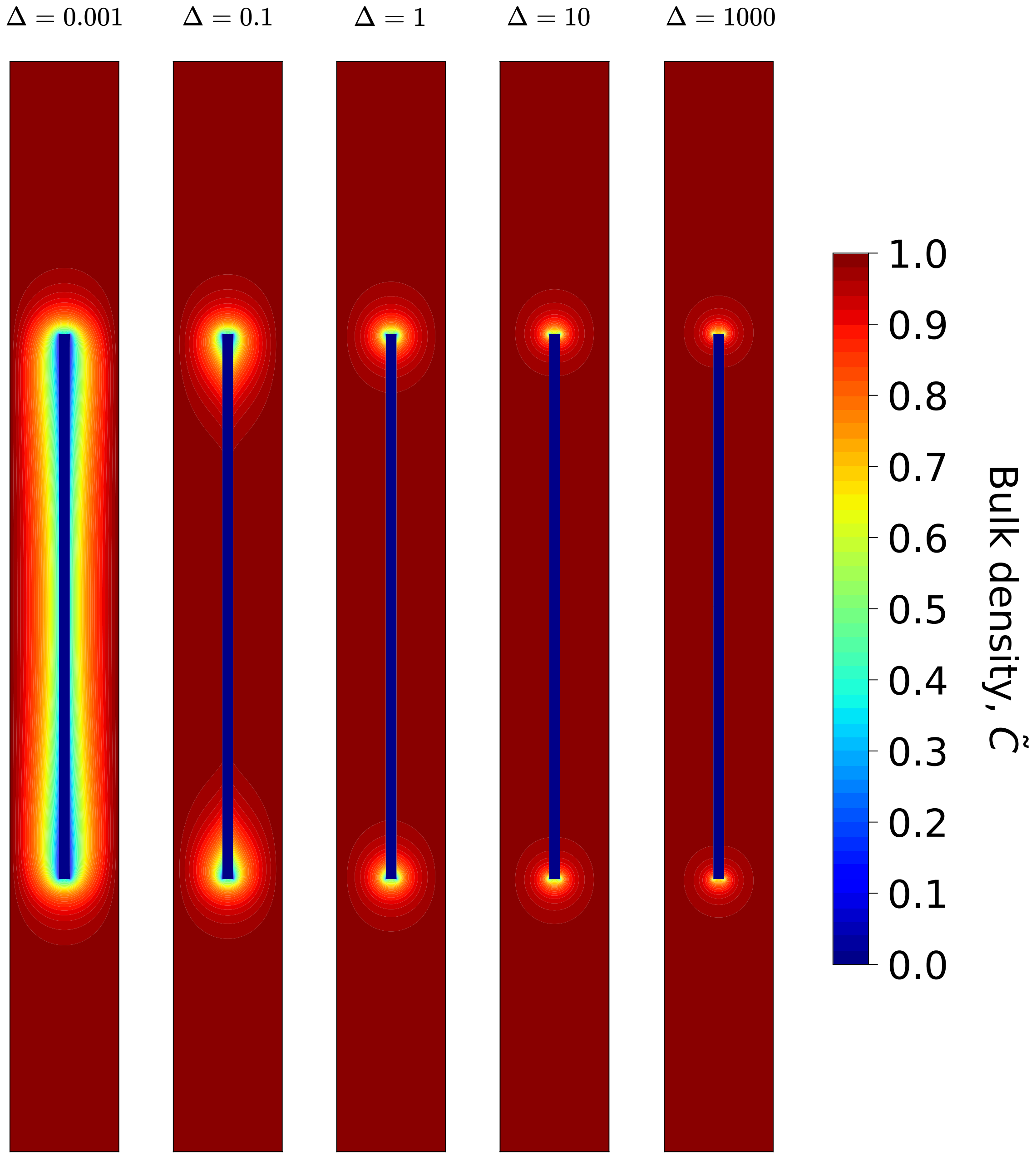

Fig. S10. Attachment rate-limited growth from bulk; diffusion-limited growth from surface: steady-state bulk density profiles ($\tilde{C}$) for a fibril with aspect ratio $\epsilon = 100$ and various Δ values.

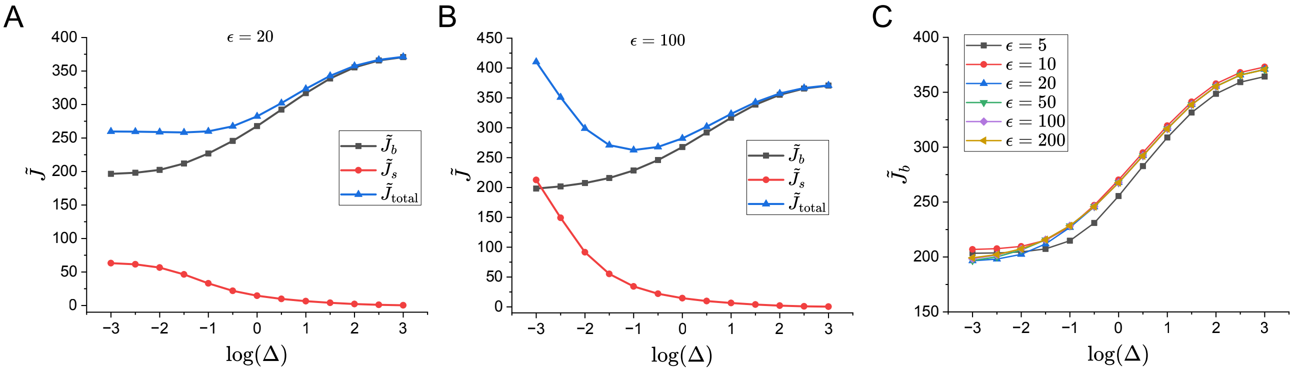

Fig. S11. Attachment rate-limited growth from bulk; diffusion-limited growth from surface: steady-state dimensionless flow rates to the tips for various Δ and ϵ values. (*A*) $\epsilon = 20$. (*B*) $\epsilon = 100$. (*C*) Dimensionless bulk flow rate $\tilde{J}_b$.

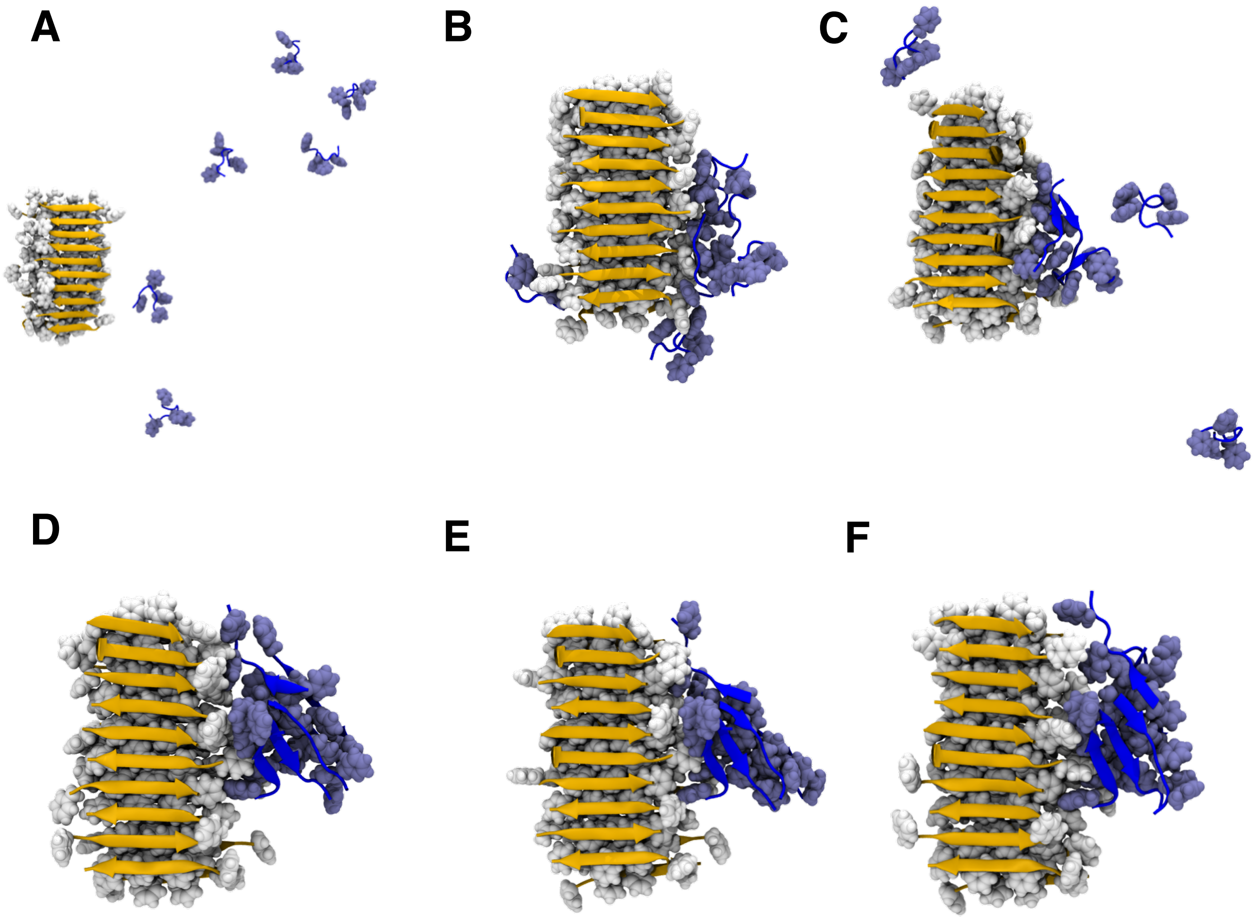

Fig. S12. Secondary nucleation for the medium-length fibril at 350 K. Visual representation of the fibril and six the peptides are displayed at (*A*) 0 ns, (*B*) 100 ns, (*C*) 200 ns, (*D*) 500 ns, (*E*) 1 μs and (*F*) 2 μs.

Movie S1. Animation of bulk-docking mechanism in fibril growth.

https://www.dropbox.com/sh/260x944qpymiiwr/AAA4M9dvXpClUsYlyT2T0QDia?dl=0

Movie S2. Animation of surface-docking mechanism in fibril growth.

https://www.dropbox.com/sh/260x944qpymiiwr/AAA4M9dvXpClUsYlyT2T0QDia?dl=0

Movie S3. Animation of short fibril at 350 K.

https://www.dropbox.com/sh/260x944qpymiiwr/AAA4M9dvXpClUsYlyT2T0QDia?dl=0

Movie S4. Animation of long fibril at 350 K.

https://www.dropbox.com/sh/260x944qpymiiwr/AAA4M9dvXpClUsYlyT2T0QDia?dl=0

Movie S5. Animation of secondary nucleation on fibril surface.

https://www.dropbox.com/sh/260x944qpymiiwr/AAA4M9dvXpClUsYlyT2T0QDia?dl=0

References

1. D Ernst, J Köhler, Measuring a diffusion coefficient by single-particle tracking: statistical analysis of experimental mean squared displacement curves. *Phys. Chem. Chem. Phys.* **15**, 845–849 (2013).
2. I Poudyal, NP Adhikari, Temperature dependence of diffusion coefficient of carbon monoxide in water: A molecular dynamics study. *J. Mol. Liq.* **194**, 77–84 (2014).
3. S Samantray, F Yin, B Kav, B Strodel, Different force fields give rise to different amyloid aggregation pathways in molecular dynamics simulations. *J. Chem. Inf. Model.* **60**, 6462–6475 (2020).
